# Construction of a high-quality genomic BAC library for Chinese peanut cultivar Zhonghua 8 with high oil content

**DOI:** 10.1186/1999-3110-55-8

**Published:** 2014-01-19

**Authors:** Yu-Ning Chen, Wen-Hui Wei, Xiao-Ping Ren, Xue-Ya Zhao, Xiao-Jing Zhou, Li Huang, Xing-Chun Tang, Hui-Fang Jiang

**Affiliations:** 1 Oil Crops Research Institute of the Chinese Academy of Agricultural Sciences/Key Laboratory of Biology and Genetic Improvement of Oil Crops, Ministry of Agriculture, Wuhan, 430062 China; 2 School of Life Sciences, Hubei University, Wuhan, 430062 China

**Keywords:** *Arachis hypogaea* L, BAC library, Clone stability, Contamination rate, Empty-vector rate, Insert sizes

## Abstract

**Background:**

*Arachis hypogaea* L. (2n = 4× = 40, AABB) is one of the most important oil and economic crop plants in the word. This species has the largest genome size of about 2,813 Mb among the oil crop species. Zhonghua 8 is a peanut cultivar planted widely in central China and has several superior traits including high oil content, high yield and disease resistance. A high-quality BAC library of Zhonghua 8 was constructed for future researches on the genomics of Chinese peanut cultivars.

**Results:**

A *Hin* d III-digested genomic BAC (bacterial artificial chromosome) library was constructed with the genomic DNA from leaves of Zhonghua 8. This BAC library consists of 160,512 clones and the average insert is estimated about 102 kb ranging from 30 to 150 kb. The library represents about 5.55× haploid genome equivalents, and provides a 99.71% probability of finding specific genes. The empty-vector rate is under 5 percent detected from 200 randomly selected clones. Probing of 384 clones with the *psbA* gene of barley chloroplast and the *atp6* gene of rice mitochondrion indicated that the contamination with organellar DNA is insignificant. Successive subculture of three clones showed that the inserts are stable in one hundred generations.

**Conclusions:**

This study presented the construction of a high-quality BAC library for the genome of Chinese cultivated peanut. Many essential experiences were summarized in the present study. This BAC library can serve as a substantial platform for development of molecular marker, isolation of genes and further genome research.

**Electronic supplementary material:**

The online version of this article (doi:10.1186/1999-3110-55-8) contains supplementary material, which is available to authorized users.

## Background

Cultivated peanut (*Arachis hypogaea* L.), a species of the genus *Arachis*, is one of the most important oilseed crops in tropical and subtropical regions of the world, mainly planted in India, China, Nigeria, Senegal, Sudan, and other countries. Its seeds contain enriched oil, protein, carbohydrate and other nutritional elements including vitamins, minerals and bioactive materials, which make peanut a major source of human nutrition, so it is very important to make peanut genetic improvement by means of molecular marker-assisted breeding and genetic engineering.

The genus *Arachis* is divided into 9 intrageneric taxonomic sections based on morphology, geographic distribution and cross compatibility (Krapovickas and Gregory 1994). Section *Arachis* is the largest section and harbors the only cultivated peanut species *A. hypogaea* that is very probably derived from a unique cross between the wild diploid species *A. duranensis* (A-genome) and *A. ipaënsis* (B-genome) (Kochert et al. 1996; Seijo et al. 2004; Burow et al. 2009). Cultivated peanut is an allotetraploid (2n = 4 × =40) with the natures of self-pollination and about 2,813 Mb genome size (Arumuganatham and Earle 1991). Its limited genetic variation has hampered the construction of high-density genetic maps and the cloning of those genes controlling the traits of interest, although several molecular genetic maps have been produced (Varshney et al. 2009; Khedikar et al. 2010; Ravi et al. 2011; Qin et al. 2012; Wang et al. 2012). Due to the lack of polymorphism at the DNA level, the crop has not been subject to marker-assisted breeding and map-based gene cloning, however, this status is being ameliorated following the development of polymorphic markers (Macedo et al. 2012). As a consequence, there is a significant need to pursue genomic strategies in cultivated peanut, with the specific goal of clarifying the genomic construction of different peanut cultivars.

Bacterial artificial chromosome (BAC) libraries, large insert genomic DNA libraries, provide a platform for physical mapping of DNA sequences, positional cloning of the genes controlling important traits, analyses of gene structure and function and genome sequencing (Pandey et al. 2012). Yüksel and Paterson (2005) constructed the first BAC library of tetraploid peanut cultivar Florunner with the trait of procumbent growth. This BAC library contained 182,784 clones with an average insert size of about 104 kb. Subsequently, Guimarães et al. (2008) developed the genomic BAC libraries of *A. duranensis* and *A. ipäensi* s. These BAC libraries have been applied in some aspects, such as isolation of desirable gene or DNA sequence (Nielen et al. 2012), BAC-end sequencing for marker development (Wang et al. 2012) and research on the comparison of these genomes, in addition to their genome sequencing.

The Chinese peanut cultivar Zhonghua 8 is a novel variety characterized by its erect growth, disease resistance to leaf spot and stunt viruses, high yield, and especially high oil content. Identification of the molecular genetic bases of these traits is expected to facilitate future peanut genetic improvements. In the present study, a high-quality BAC library of Zhonghua 8 was constructed, which represents about 5.55× haploid genome equivalents and has an average insert size over 100 kb, thus constituting a reliable tool for future researches on the genomics of Chinese peanut cultivars.

## Methods

### Plant materials

Peanut cultivar Zhonghua 8 is an erect growth genotype with short growth period (105 to 120 days from sowing to maturity), leaf spot and stunt viruses resistances, drought tolerance, high oil content (56.13%), big pod (100-pod weight 192.0 g), big seed (100-seed weight 84.2 g), high shelling percentage (75%), desirable pod and kernel features. Zhonghua 8 plants were grown under weak light in the greenhouse at the Institute of Oil Crops, Chinese Academy of Agricultural Sciences. The young etiolated leaves were washed with fresh water, then airdried at room temperature and frozen with liquid nitrogen, quickly stored at -80°C for use.

### BAC vector preparation

The CopyControl™ BAC Cloning Kit (Epicentre company) was used for construction of the library. The BAC vector was digested with *Hin* d III, dephosphorylated, and stored at -20°C in 25 ng/ul aliquots until needed.

### Preparation and digestion of high-molecular weight (HMW) DNA

The isolation of peanut nuclei was performed according to the protocols reported by Zhang et al. (1995) and Yüksel and Paterson (2005) with some modifications. 20 g of frozen leaves were ground in liquid nitrogen until fine powder was obtained, then dissolved in 200 ml of fresh extraction buffer [0.005 M citric acid, 0.5 M glucose, 0.01 M Na_2_EDTA, 2.0% (w/v) polyvinylpyrrolidone-40 (PVP-40), 5% (v/v) Triton X-100, 0.25% (w/v) spermidine, 0.1% (w/v) ascorbic acid, 0.2% (v/v) 2-mercaptoethanol, 0.1% (w/v) disodium diethylthiocarbamate (Na_2_Et_2_dtc) and 0.4% (w/v) NaHSO_3_, titrated to pH 6.5 with NaOH] on ice for about 15 min. The samples were filtered twice through two layers of cheese clothes and two layers of miraclothes. The filtrates were centrifuged for 15 min at 1600 g after a centrifuge for 15 min at 60 g. The precipitated nuclei were dissolved in extraction buffer and centrifuged at the same speed three times until the nuclei looked clean, the final wash of the nuclei was in the same extraction buffer without Triton X-100. The following steps were only followed for HMW DNA extraction: the plugs were incubated at 50°C for 24 h in lysis buffer [0.005 M citric acid, 0.14 M NaCl, 0.05 M Na_2_EDTA, 2% (w/v) PVP-40, 1% (w/v) sodium dodecyl sulfate (SDS), 1% sodium lauryl sarcosine titrated to pH 6.5 with NaOH, and autoclaved] and the same antioxidants at similar proportions as in the extraction buffer, and 0.2 mg/ml Proteinase K was added. The buffer was replaced, and plugs were incubated at the same temperature for another 24 h. The plugs were incubated at room temperature for at least 4 h in 70% ethanol then stored at -20°C until use.

The plugs were placed into T_10_E_1_ (PH = 8.0) containing 0.1 mM PMSF (Phenylmethanesulfonyl fluoride) and vortexed on ice for 1 h, repeated three times, then treated three times in T_10_E_1_ (PH = 8.0) without PMSF under the same conditions. The plugs were then digested with *Hin* d III enzyme as the method reported by Yüksel and Paterson (2005). Briefly, serial dilutions of *Hin* d III (NEB) (0, 0.1, 0.15, 0.2, 0.3, 0.5, 0.75, 1, and 2 U per milligram of plug) were added to the samples and the samples incubated at 4°C for 4 h. Partial digestion was carried out at 37°C for 7 min, then 0.5 M EDTA was added to the tubes to stop the reactions. The partially digested samples were resolved on 1% agarose gels run in 0.5 × TBE buffer by pulsed field gel electrophoresis (PFGE) at 6 V/cm, with 1- to 40-s switch times and a linear ramp, for 18 h. Optimum enzyme concentration was determined by visualizing maximum fragment concentration in the 100- to 300-kb range.

### Construction of BAC library

For ligation, a constant 25 ng of vector was used, and varying amounts of insert ranging from 60 to 120 ng were tested for each size selection. The vector/insert (V/I) ratio, which gave the best efficiency and average insert size, was chosen. Ligation reactions were performed in 60 μl volumes and incubated at 16°C for 10 h. After desalting, 2–3 μl of reactions were transformed into *Escherichia coli* EP1300 (Epicentre) competent cells by electroporation (BioRad Gene Pulser® II Electroporation System). For electroporation, 1.25 kV and 200× of resistance were used. The electroporated cells were immediately mixed with 1 ml of SOC media and grown for 1 h at 37°C before separation on selective medium (LB medium) with 12.5 μg chloroamphenicol, 0.55 mM IPTG, and 80 μg/ml X-gal. After 18 h of incubation at 37°C, a sampling of 10–20 colonies were picked and tested. Randomly selected white colonies were inoculated into 1 ml LB CM liquid medium and grown for 16 h at 37°C. The liquid cultures were subjected to minipreps by an alkaline lysis protocol, and the DNA was digested with 10 U of *Not* I (NEB) for 4 h at 37°C. The digested samples were resolved on 1%, 0.5 × TBE agarose by PFGE with the following parameters: 3- to 20-s linear ramp, 6 V/cm, and a 16-h run time. The ligation reactions with average insert size of 100 kb or more were mass-transformed, plated, and directly picked with toothpicks into 384-well plates. The clones were replicated three times with Replicator and stored in FM medium [LB + 36 mM K_2_HPO_4_, 13.2 mM KH_2_PO_4_, 1.7 mM sodium citrate, 0.4 mM MgSO_4_, 6.8 mM (NH_4_)_2_SO_4_, and 4.4% glycerol] at -80°C.

### Insert-size characterization

200 clones were randomly picked out for insert-size characterization. The *Not* I-digested clones were detected on 1%, 1 × TAE agarose by PFGE with the following parameters: 5- to 15-s linear ramp, 6 V/cm, and a 16-h run time.

### Detection of organelle DNAs contamination

The BAC clones were dotted on N^+^ nylon filter with Replicator, and then conversely cultured on LB medium containing chloramphenicol at 37°C overnight. The clone carrying empty BAC vector was used as a negative control, the clones containing the *psbA* gene of barley chloroplast and the *atp6* gene of rice mitochondrion, respectively, were used as positive controls. The filters were treated under following sequential conditions: 10% SDS, denaturing solution, neutralizing solution, neutralizing solution, 2xSSC, 0.1% SDS, 2xSSC, 5 min, respectively, then 0.4 N NaOH 20 min. The sequential washing of filters was performed with 0.1% SDS (in 5xSSC) for 20 min twice and 2xSSC for 10 min twice, then the filters were dried at 80°C for 2 h.

1 μL (25 ng) probe DNA mixed with 2 μL primers and 11 μL water was denatured at 95°C for 3 min, placed on ice for at least 10 min, then 2.5 μL 10 × Buffer, 2.5 μL dNTP Mixture, 5 μL α-^32^P dCTP and 1 μL Klenow enzyme were added into the probe solution, the labeling was performed at 37°C for 1–2 h, subsequently the mixture was denatured again at 95°C for 3 min, placed on ice until for use. The filter was pre-hybridized at 68°C for 3 h, then 5 μL labeled probes each were added into the pre-hybridization solution (6 × SSC, 0.05 × blotto, 0.5% SDS, 100 μg/ml ssDNA), the hybridization was performed at 68°C for 16 h. Finally, the hybridized filters were washed twice with solution I (2xSSC, 0.1% SDS) at room temperature for 5–10 min, then washed twice with solution II (1xSSC, 0.1% SDS) at 68°C for 2 h, and imaged and quantitatively analyzed using a Biorad FX molecular imager.

### Detection of BAC clone stability

Three BAC clones were randomly picked out for successive cultures; the clones of the 100^th^ generation could be obtained on the fifth day since *E. coli* propagated 20 generations each day (Sambrook and Russell 2001). The BAC clones of the first day and the fifth day were digested with *Not* I to analyze their stability.

## Results

### Production of BAC library

Eleven successful ligation reactions were performed for construction of the BAC library. The numbers of obtained clones were different for each ligation, 22, 105 clones were obtained in the third ligation reaction, and only 5, 720 clones could be picked out from the seventh ligation reaction, which was probably related to ligation, transformation and DNA quality. A total of 160,512 clones were obtained from the eleven successful ligation reactions, they were deposited in 418 384-well plates.

### Detection of insert size

**Figure 1:**
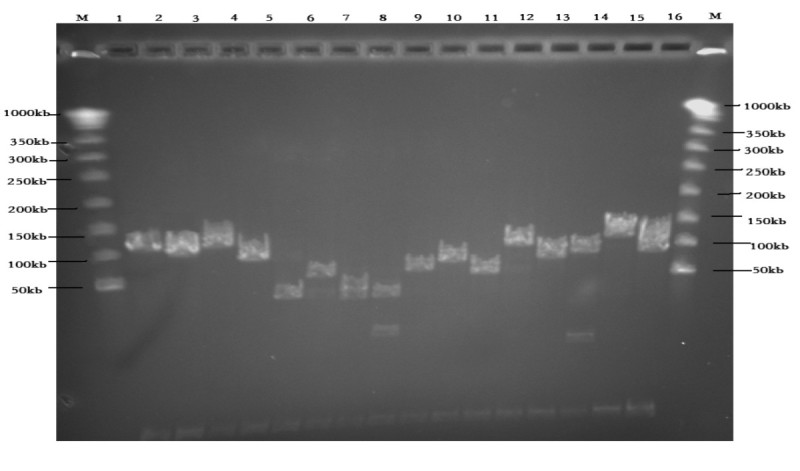
**Detection of insert size.** M: MidRange I PFG Marker; 1–16: BAC clone samples from one ligation reaction.

**Figure 2:**
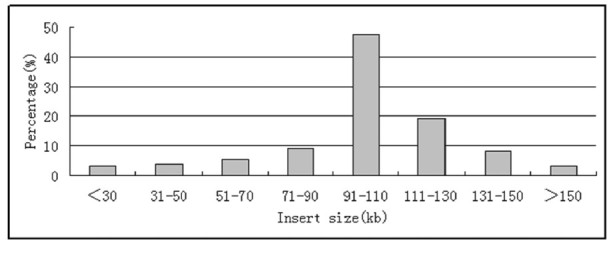
**Distribution of insert size.**

200 clones randomly selected from the eleven ligation reactions were digested with *Not* I. The PAGE results showed an insert size of about 102 kb with a range of 30 to 150 kb (Figures 1 and 2). Parts of digested BAC clones presented two bands, their insert sizes should be the sum of two band sizes (Figure 1). Only seven empty BAC vectors were detected in the 200 randomly selected BAC clones, so the empty-vector rate was estimated to be less than 5 percent. The *A. hypogaea* haploid genome size is about 2.831 × 10^9^ bp, the library represents about 5.55× haploid genome equivalents. As the formula N = ln(1-p)/ln(1-f) (N, the clone number of library; p, the probability of a gene cloned; f, insert size/genome size) reported by Peterson et al. (2002), this library provides a 99.71% probability of finding any specific genes.

### Detection of organelle DNAs contamination

**Figure 3:**
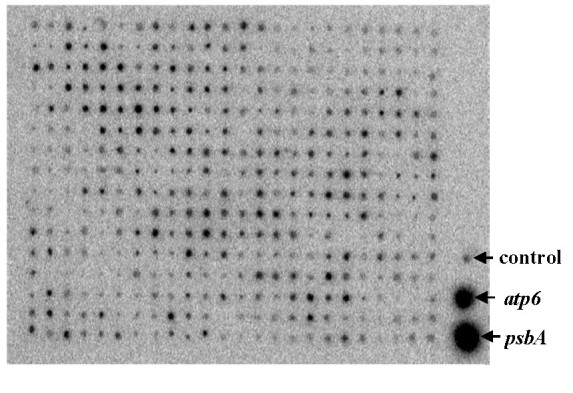
**Detection of organelle DNAs contamination by colony hybridization.** *psbA*: the clone carrying the *psbA* gene of barley chloroplast; *atp6*: the clone carrying the *atp6* gene of rice mitochondrion; control: the clone carrying empty BAC vector.

The colony hybridization results showed that no organelle DNAs could be detected in the 384 clones randomly selected from the eleven ligation reactions. Figure 3 presented the hybridization results of 384 BAC clones showed that the intensity of the hybridization signals of all the BAC were consistent with that of the negative control. The results indicated that the rate of organelle DNAs contamination was at least very low.

### Detection of insert stability

**Figure 4:**
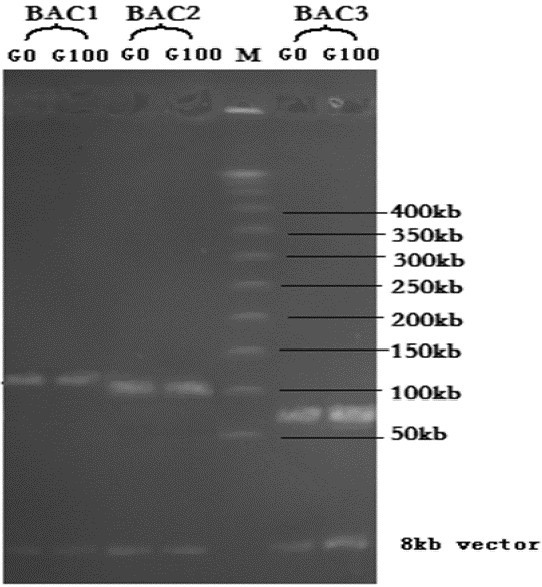
**Detection of clone stability.** G0: the zero generation; G100: the 100^th^ generation; M: MidRange I PFG Marker.

The insert sizes of individual clones were consistent with each other at the zero generation and the 100^th^ generation (Figure 4), which showed that the BAC clones were stable in the *E. coli* cell at least in one hundred generations. Thus, the BAC library can be conserved at ultra-low temperature for long-term use.

## Discussion

The quality of a BAC library is reflected by the insert size, empty-vector rate, organelle DNAs contamination rate, insert stability and genome coverage. The present results indicated that this BAC library is of high quality, and is sufficient for the target gene isolation and for the genomics research of Chinese *A. hypogaea* cultivar.

The leaf of *A. hypogaea* is enriched with polysaccharides and phenolic compounds. The polysaccharides could result in the addition of the viscosity of leaf extraction and the delay of DNA filtration. The phenolic compounds were easily oxidized, the oxides could combine with DNA and form colloidal compounds. So, these two extractions could result in the degradation of HMW DNA. In the present study, two efficient strategies were adopted to weaken the interfering of polysaccharides and phenolic compounds with HMW DNA, one was to add PVP-40 into cell lysis buffer to suppress the oxidation of phenolic compounds (Yüksel and Paterson 2005), the other was to grow the plants under weak light to reduce the synthesis of polysaccharides and chlorophyll.

Sometimes HMW DNA could not be efficiently digested by *Hin* d III enzyme for the recovery of 100–300 kb DNA segments, which was probably caused by the following reasons: firstly, the histones in the chromosomes were not completely digested, the *Hin* d III enzyme could access DNA to cut it, to solve this problem, we should prolong the digestion time to 15 min or add the concentration of proteinase K to 0.5 mg/ml; secondly, proteinase K was not completely inactivated by PMSF, thus proteinase K could inactivate *Hin* d III enzyme, at this situation, the plugs should be washed time after time by TE solution containing PMSF; thirdly, the rudimental PMSF also could inactivate *Hin* d III, so the plug should be adequately washed by TE solution without PMSF following the washing with TE solution containing PMSF.

At the start of BAC library construction, an unqualified ligation reaction mainly produced 40–60 kb inserts (results not shown), the reason is probably that a number of short digested segments were wrapped with large segments together, then the short segments were preferentially ligated to BAC vector. This problem was effectively solved in subsequent ligation reactions. The recoveries of 100–300 kb segments were separated again by PFGE to remove the short segments and to recover the large ones. At the same time, other effective steps were to reduce the concentration of HMW DNA wrapped in the plugs and to break the plugs placed in the sample holes, these strategies could promote the separateness of DNA segments with different size.

The ligation and transformation are two crucial steps during the construction of BAC library. The final recovered LMP agarose gel blocks embedding DNA should be eluted to recover DNA in dialytic-bag by PFGE before ligation, the elution solution dissolving DNA was then directly used as ligation system, so the concentration and purity of DNA dissolved in the elution solution are crucial for next ligation reaction. To satisfy these needs, the electrophoresis buffer must be prepared with sterilized ultrapure water, the eluted DNA should be quantitatively analyzed to satisfy the optimal ratio between DNA and BAC vector, and the DNA should be ligated with the BAC vector immediately. For increasing the transformation efficiency, the DNA ligase should be inactivated at 65°C after the ligation reaction was finished, the ligation product need to be desalted by dialysis, and transformed immediately.

This BAC library will be a substantial platform for the isolation of novel genes especially associated with oil content, the screening of BAC clones identifying specific chromosome (Wang et al. 2010; Yan et al. 2012), the isolation of characteristic repeat sequences (Li et al. 2010; Nielen et al. 2012), the exploitation of SSR marker for the construction of physical and genetic maps (Qin et al. 2012), and for the study of the genome re-sequencing of Chinese peanut cultivars.

## Conclusions

In general, this study presented the construction of a high-quality BAC library for the genome of Chinese cultivated peanut. Many essential experiences were summarized in the present study. These experiences will be valuable for the production of *Arachis* or other plant BAC libraries.

## Authors’ original submitted files for images

Below are the links to the authors’ original submitted files for images. Authors’ original file for figure 1 Authors’ original file for figure 2 Authors’ original file for figure 3 Authors’ original file for figure 4
