# Supplementary figures and images for: Construction of a high-quality genomic BAC library for Chinese peanut cultivar Zhonghua 8 with high oil content
Source: Bot Stud. 2014 Jan 19;55:8. doi: 10.1186/1999-3110-55-8 (PMC5432765; doi:10.1186/1999-3110-55-8)

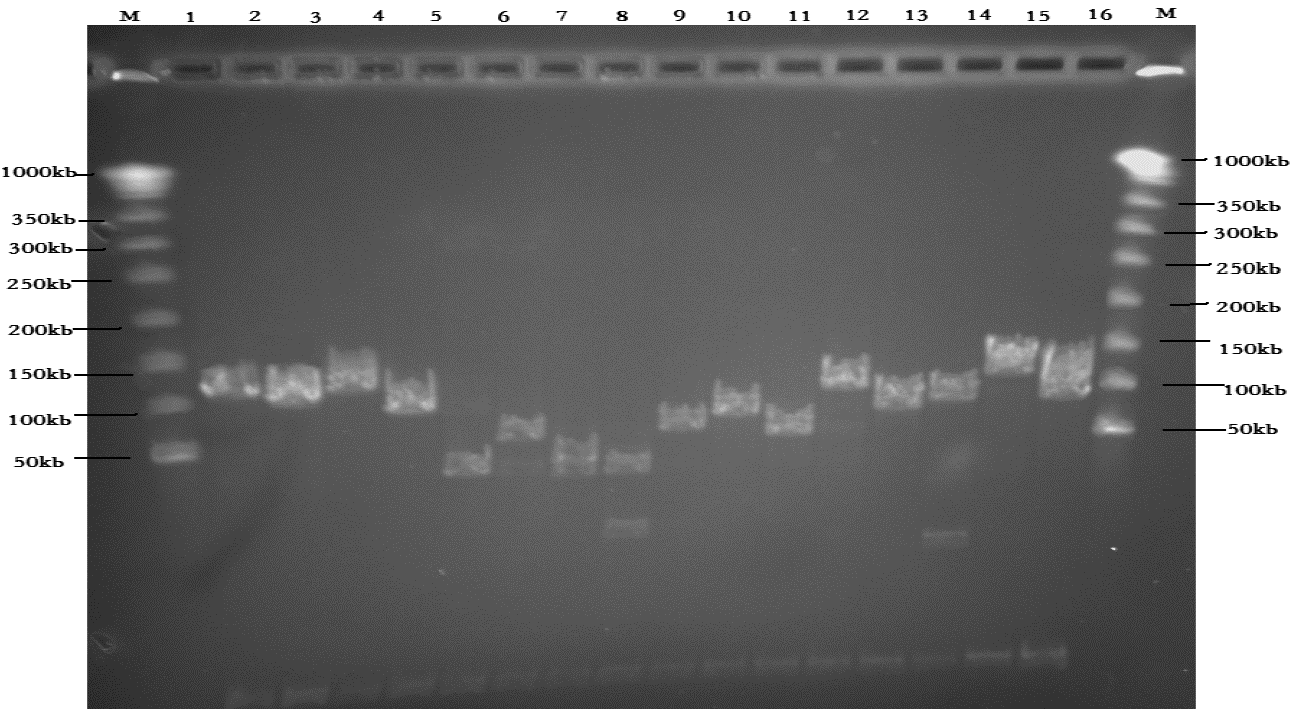

Supplement: Supplementary file 1 — Authors’ original file for figure 1 [file 40529_2012_83_MOESM1_ESM.tiff]

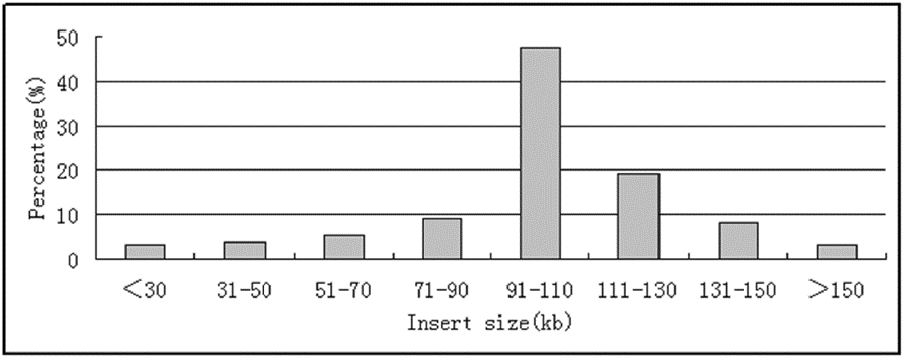

Supplement: Supplementary file 2 — Authors’ original file for figure 2 [file 40529_2012_83_MOESM2_ESM.tiff]

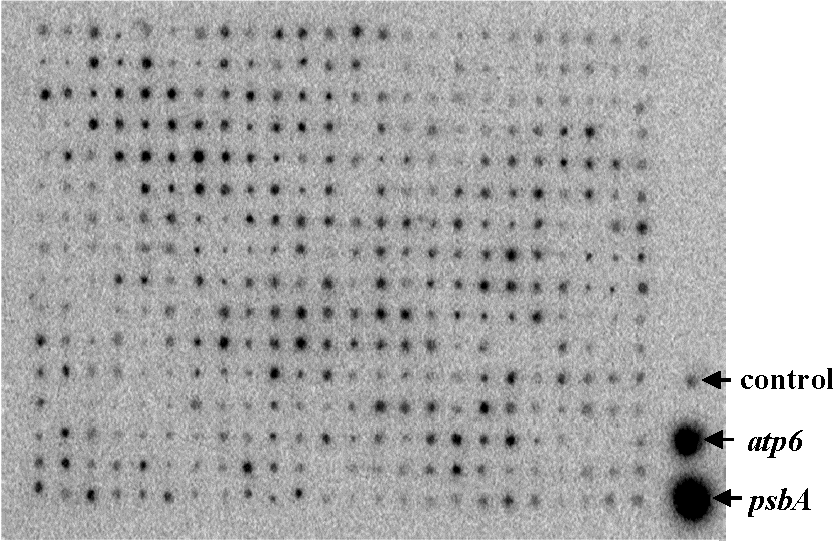

Supplement: Supplementary file 3 — Authors’ original file for figure 3 [file 40529_2012_83_MOESM3_ESM.tiff]

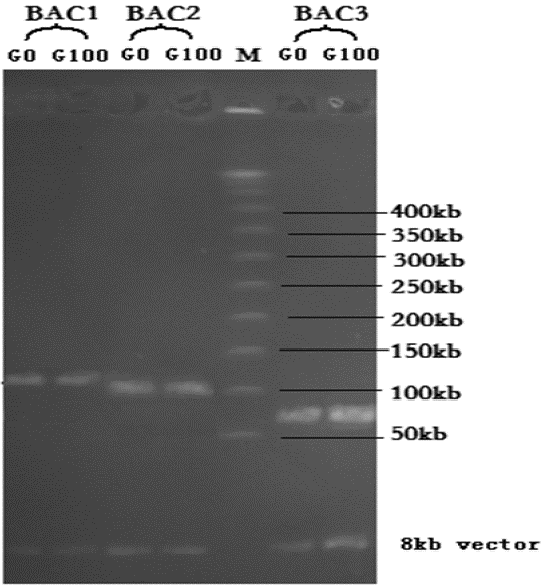

Supplement: Supplementary file 4 — Authors’ original file for figure 4 [file 40529_2012_83_MOESM4_ESM.tiff]
